# Early detection and prediction of cardiotoxicity after radiation therapy for breast cancer: the BACCARAT prospective cohort study

**DOI:** 10.1186/s13014-016-0627-5

**Published:** 2016-04-07

**Authors:** Sophie Jacob, Atul Pathak, Denis Franck, Igor Latorzeff, Gaelle Jimenez, Olivier Fondard, Matthieu Lapeyre, Daniel Colombier, Eric Bruguiere, Olivier Lairez, Benoit Fontenel, Fabien Milliat, Radia Tamarat, David Broggio, Sylvie Derreumaux, Marianne Ducassou, Jean Ferrières, Dominique Laurier, Marc Benderitter, Marie-Odile Bernier

**Affiliations:** Institut de Radioprotection et de Sureté Nucléaire (IRSN), PRP-HOM, SRBE, LEPID, Fontenay-aux-Roses, France; Clinique Pasteur, Unité d’Hypertension artérielle, facteurs de risque et insuffisance cardiaque, Toulouse, France; Clinique Pasteur, Radiothérapie (Oncorad), Toulouse, France; Clinique Pasteur, Cardiologie générale et interventionnelle, Toulouse, France; Clinique Pasteur, Radiologie, Toulouse, France; University Hospital Rangueil, Cardiologie B, Toulouse, France; Clinique Pasteur, Laboratoire d’analyse, Toulouse, France; Institut de Radioprotection et de Sureté Nucléaire (IRSN), PRP-HOM, SRBE, L3R, Fontenay-aux-Roses, France; Institut de Radioprotection et de Sureté Nucléaire (IRSN), PRP-HOM, SRBE, LR2I, Fontenay-aux-Roses, France; Institut de Radioprotection et de Sureté Nucléaire (IRSN), PRP-HOM/SDI/LEDI, Fontenay-aux-Roses, France; Institut de Radioprotection et de Sureté Nucléaire (IRSN), PRP-HOM, SER, UEM, Fontenay-aux-Roses, France; INSERM, University Paul Sabatier, UMR1027, Epidemiology of cardiovascular diseases, Toulouse, France; Institut de Radioprotection et de Sureté Nucléaire (IRSN), PRP-HOM, SRBE, Fontenay-aux-Roses, France

**Keywords:** Breast cancer, Radiotherapy, Cardiac toxicity, Functional and anatomical cardiac imaging, Biomarkers, Heart dosimetry

## Abstract

**Background:**

Radiotherapy (RT) for breast cancer presents a benefit in terms of reducing local recurrence and deaths resulting from breast cancer but it can lead to secondary effects due to the presence of neighboring cardiac normal tissues within the irradiation field. Breast RT has been shown to be associated with long-term increased risk of heart failure, coronary artery disease, myocardial infarction and finally cardiovascular death more than 10 years after RT. However, there is still a lack of knowledge for early cardiotoxicity induced by breast RT that can appear long before the onset of clinically significant cardiac events. Based on a 2-year follow-up prospective cohort of patients treated with breast RT, the BACCARAT (BreAst Cancer and CArdiotoxicity Induced by RAdioTherapy) study aims to enhance knowledge on detection and prediction of early subclinical cardiac dysfunction and lesions induced by breast RT and on biological mechanisms potentially involved, based on functional and anatomical cardiac imaging combined with simultaneous assessment of multiple circulating biomarkers and accurate heart dosimetry.

**Methods/Design:**

BACCARAT study consists in a monocentric prospective cohort study that will finally include 120 women treated with adjuvant 3D CRT for breast cancer, and followed for 2 years after RT. Women aged 50 to 70 years, treated for breast cancer and for whom adjuvant 3D CRT is indicated, without chemotherapy are eligible for the study. Baseline (before RT) and follow-up data include measurements of functional myocardial dysfunction including strain and strain rate based on 2D-speckle tracking echocardiography, anatomical coronary lesions including description of plaques in segments of coronary arteries based on Coronary computed tomography angiography, and a wide panel of circulating biomarkers. The absorbed dose is evaluated for the whole heart and its substructures, in particular the coronary arteries. Analysis on occurrence and evolution of subclinical cardiac lesions and biomarkers will be performed and completed with dose-response relationship. Multivariate model of normal tissue complication probability (NTCP) will also be proposed.

**Discussion:**

Tools and results developed in the BACCARAT study should allow improving prediction and prevention of potential lesions to cardiac normal tissues surrounding tumors and ultimately enhance patients’ care and quality of life.

**Trial registration:**

ClinicalTrials.gov: NCT02605512

## Background

It has been shown that adjuvant Radiotherapy (RT) for breast cancer presents a benefit in terms of reducing local recurrence and deaths resulting from breast cancer [1]. However, breast RT can lead to secondary effects due to the presence of neighboring normal tissues within the irradiation field, including the heart. The severity of radiation-induced toxicity to healthy tissues can unfortunately affect the quality of life of cancer survivors.

### Long term cardiac events

Breast cancer RT irradiation of the heart has been shown to be associated with long-term cardiac toxicity such as heart failure, coronary artery disease, myocardial infarction and finally cardiovascular death more than 10 years after RT with relative risks within the range of 1.2 to 3.5 by comparing left breast treated patients (with higher exposure to heart) to right ones or unexposed ones [1–4]. Moreover, cardiac damage was shown to be correlated with the heart-absorbed dose with 7.4 % rate increase of ischemic heart disease per one Gray (95 % confidence interval, 2.9 to 14.5; *P* < 0.001), with no minimum threshold for risk [4]. Retrospective studies based on records of patients treated with radiation therapy for breast cancer who had undergone a coronary angiography many years after RT, also showed a link between radiation and location of stenosis as stenosis were often present in left anterior descending artery [5–7]. These studies revealed the importance of simultaneous consideration of the location of radiation doses at the structures of the heart combined with localized effects, particularly in the coronary arteries [8, 9]. However, these retrospective studies only considered patients treated until the 90’s that secondarily developed cardiac diseases but didn’t provide information on early signs of cardiotoxicity that can appear long before the onset of these clinically significant cardiac events.

### Subclinical cardiac changes

Long before the onset of clinically significant cardiac events occurring many years after RT, some subclinical cardiac changes can occur over weeks, months or first years after RT, that can be detected either based on functional dysfunction or anatomical modifications measurements.

#### Echocardiography and myocardial changes

Cardiac echography can be used to evaluate myocardial dysfunction. Global Longitudinal Strain and strain rate (GLS) assessed using automated 2D-speckle-tracking echocardiography (STE) is a recent technique for detecting and quantifying subtle disturbances in Left ventricular (LV) systolic function. This technique is operator independent, more reproducible than ejection fraction (EF) and GLS was shown to be a superior predictor of cardiac events compared to EF. In particular, this method was used in the context of cardiotoxicity after breast RT. In Belgium, two prospective studies were based on measurements of strain and strain rate as an indicator of myocardial contractility [10, 11]. Comparing the strain before breast radiation, then a few months later, both studies showed significant changes related to radiotherapy. The most recent study included 51 women treated for left breast cancer and 24 women treated for right breast cancer. Both strain and strain rate were significantly decreased (mean 5 %) during the first year following RT for left-sided patients: a decrease in strain was observed at all post-RT time points (−17.5 ± 1.9 % immediately after RT, −16.6 ± 1.4 % at 8 months and −17.7 ± 1.9 % at 14 months vs −19.4 ± 2.4 % before RT, *p* < 0.01). Similar results for longitudinal strain decrease were also observed in a recent German study [12]. Based on this clinical state, it appeared that early cardiotoxicity of radio-induced breast RT could be measured based on subclinical myocardial functional changes which is concordant with recent publication of recommendations in the evaluation of cardiovascular complications after radiotherapy in adults including in particular cardiac echography examination [13]. However these results on strain and strain rate remain to be confirmed and more precise analyses depending on the dose distribution over the surface of myocardial could enable a better understanding of a possible dose response.

#### CCTA and coronary changes

Coronary computed tomography angiography (CCTA) provides morphological information: visualization of the coronary arteries; visualization of calcification of the coronary arteries and determination of calcium score which reflects the evolution of the coronary disease may help to visually locate vulnerable lipid-rich soft plaques (little or non-calcified) as potential risk of coronary occlusion. By limiting radiation exposure to the phase of interest (generally, the motion sparse diastolic phase), the radiation dose can be reduced, with an average dose < 5 mSv [14]. This CCTA method was used for patients treated with radiation therapy to the chest for Hodgkin lymphoma that developed coronary damages after their RT [15, 16] but even more generally in the follow-up of cardiac patients [17]. Lehman et al. showed the potential of CCTA for accurate monitoring of the progression of calcified and non-calcified plaques from a population of 69 patients with chest pain examined twice at 2-year intervals [17]. From tracking nearly 9000 segments of coronary arteries in the cohort, a significant 12.7 % increase in the mean number of cross-sections containing any plaque was observed (*p* = 0.01). While this study did not consider radiation exposure of the heart, it does illustrate that the CCTA could be used for monitoring short-term changes in coronary changes. No study focused on women treated with breast radiotherapy while CCTA has a potential to detect the onset or progression of early coronary changes due to irradiation.

### Circulating biomarkers

From a biological point a view, understanding the biological mechanism of the initiation and progression of early radiotherapy side effects on normal tissue such as cardiac tissue is also an important issue. To our knowledge, there are no specific and early biomarkers of radiation-induced heart damage at the present time. According to the dose distributions at heart, ionizing radiation can generate cardiac injury and a major challenge remains the identification of reliable biomarkers that could help to diagnose and predict cardiotoxicity. Depending on the type of injuries (microvascular rarefactions, coronary damage, tissue inflammation) potentially relevant biomarkers are different.

Many classical biomarkers (*C-reactive protein*, *N-terminal pro-B–type natriuretic peptide* (NT-proBNP) and *troponin* (TnI), *…*) were shown to be potential biomarkers for cardiac damage, in particular after radiotherapy [18–20]. Circulating inflammatory cytokins [21] can also sign tissue inflammation. It was also showed that irradiation induces acute endothelial activation or dysfunction that can be observed many weeks after irradiation and resulting in pro-inflammatory endothelial phenotype.

Another hypothesis to explore is that the irradiation may be responsible for an increase in the circulating levels of certain miRNAs expressed by cells of the heart tissue Currently, the potential function of extracellular miRNAs is being studied intensively, and the first studies have confirmed that miRNAs may indeed function in cell-to-cell communication. [22]. Many studies have reported the use of miRNAs as circulating biomarkers for diagnosis or prognosis of cardiovascular diseases. Although many of those studies still require replication in multiple independent study populations, the picture emerges that some plasma miRNAs are quite specific for cardiovascular pathologies and may be useful for diagnostic and monitoring purposes.

The complex and multifactorial nature of atherogenesis and development of atherothrombotic complications involves numerous interactions between various cell types inside the vascular wall (e.g., macrophages and smooth muscle cells) and in the blood (e.g., leukocytes and platelets). One relatively recent advance in this area is the discovery of circulating microparticles. High levels of circulating microparticles found in many cardiovascular diseases demonstrate the importance of platelet, monocyte and endothelial activation and could condition remote sustainability illnesses [23].

Based on this state of art, the simultaneous assessment of multiple biomarkers may be of strong interest for RT cardiotoxicity.

### Cardiac radiation exposure

Considering the dosimetric point of view is also important to quantify precisely the risk of cardiac lesion. As awareness of radiotherapy cardiotoxicity has grown and technology has developed, in the history of breast cancer RT regimens, the range of doses to the heart has changed over the past few decades [24–26]. Mean heart dose decreased from more than 5Gy in the 50’s to less 3 Gy in the last decade [27] and availability of 3-dimensional CT planning (3D CRT) in the 90’s has enabled clinicians to reduce normal tissue doses generally.

Routinely, whole heart dose-volume histograms (DVH) are available based on radiotherapy simulation CT scans. However these DVH cannot provide information on the location of higher doses. Highest cardiac radiation doses can be observed in the apex and the apical-anterior segment and some hot spots > 50Gy persist in some parts of the heart [12] which may still today induce cardiac lesions, in particular for coronaries [28]. To avoid damage and complications to healthy tissues, dose constraints are applied to the organs at risk. The QUANTEC - Quantitative Analysis of Normal Tissue Effects in the Clinic- recommendations, specify that the heart should always be contoured and less than 10 % of the heart must receive more than 25 Gy: "For partial irradiation, a conservative model-based estimates predict that a V25Gy < 10 % (in 2 Gy per fraction) will be associated with a <1 % probability of cardiac mortality 15 years after RT." [29]. However, no dose recommendation exists for the coronary arteries whereas calculated mean heart doses highly differ from mean coronary arteries doses due to important dose gradient. In clinical practice, coronary arteries are not contoured. The specific relationships between doses to cardiac structures and subsequent toxicity have not been well defined. As a consequence, studies based on radiation dosimetry that is able to take account of the distribution of dose within the heart (rather than just the mean heart dose) may provide further insight into the spatial location of heart damages in breast cancer RT [28]. Precise approach of heart substructure dosimetry has been developed at IRSN for Hodgkin patients by merging RT simulation CT scans and CCTAs to obtain detailed cardiovascular anatomies and finally radiation treatment parameters were used to estimate CA radiation doses [30]. Similar approach could be used for breast cancer radiotherapy.

### NTCP models

The relationship between dosimetry data and toxicity to healthy tissue is a key element in the ability to calculate risk. Mathematical models have been developed in recent years with the aim of using dosimetric data (mainly DVH) to estimate a complication probability (mainly a clinical sign): the NTCP models (Normal Tissue Complication Probability). Research on parameters to optimize the NTCP models (physical, imaging, dosimetry, clinical) are a major challenge to improve knowledge on the relationship between a received dose and toxicity to healthy tissue [31, 32]. In addition, the enrichment with biological data of these models is a fundamental NTCP track in the overall goal of optimizing risk prediction. In the context of cardiotoxicity, this type of model has been poorly exploited [33], due to the long lag time to see the clinical signs and the fact that toxicity evaluation criteria is limited primarily to acute effects, but rarely to the late effects that develop after several years. By defining a short term subclinical cardiotoxicity, it would nevertheless be possible to apply these methods to know predictors of subclinical complications and ultimately to predict the risk of subclinical complication itself predictive of clinically significant complication that would occur several years later.

## Methods/Design

### Study objectives

#### General aim

Based on a 2-year follow-up prospective cohort of patients treated with breast radiotherapy (3D CRT), the BACCARAT study aims to enhance knowledge on detection and prediction of early subclinical cardiac dysfunction and lesions induced by breast cancer radiotherapy and on biological mechanisms potentially involved, based on functional and anatomical cardiac imaging combined with simultaneous assessment of multiple circulating biomarkers and accurate heart dosimetry.

#### The primary objective is:

I. To evaluate the occurrence and evolution of subclinical cardiac lesions at myocardial levels (measure of myocardial contractility - strain) and coronary levels (measure of coronary plaque index) based on cardiac imaging techniques (cardiac ultrasound exam"2D strain" and CCTA).

#### The secondary objectives are:

II. Study of the temporal variations of a panel of targeted circulating biomarkers of microvascular rarefactions, coronary damage, and tissue inflammation related with breast RT.
III. Evaluate precisely the absorbed doses, for the whole heart and for the different structures of the heart, specifically the coronary arteries.
IV. Analyse the dose-response relationship between subclinical cardiac changes, biomarkers, and absorbed doses and propose multivariate model of normal tissue complication probability (NTCP), which takes into account relationships among all these parameters and potentially offer a powerful approach to the optimization of risk assessment.
V. Establish a biobank of plasma for further analysis of biomarkers linked to the study of RT cardiotoxicity.

### Study design and population

BACCARAT study consists in a monocentric prospective cohort study that will finally include 120 women treated with adjuvant 3D CRT for unilateral breast cancer in the Clinique Pasteur in Toulouse, France, without chemotherapy, and followed for 2 years after RT.

In the Clinique Pasteur, there are no competing methods to 3D-CRT at the moment. All patients with breast cancer are treated with 3D-CRT and breath-hold gating is used for patients treated left breast cancer with heart very close to the anterior chest wall or for dose constraints achievement (mean heart dose < 5Gy and V25Gy < 10 %). Women aged between 50 and 70 years, for whom adjuvant 3D CRT is indicated with or without breath-hold gating, with no indication of chemotherapy are eligible for the study. Both women with left (80 % of the sample) or right (20 % of the sample) breast cancer are included. This choice to include both sides is justified by two reasons: (i) if the internal mammary chain is treated significant doses can result, whatever the treated side; (ii) if the internal mammary chain is not treated the right side treatment will provide lower doses, an important point for the dose response analysis. With this design, each patient (after RT) could be her own control (before RT). Moreover, the left-sided patients could also be compared to the right-sided patients. Inclusion and exclusion criteria are presented in Table 1.

**Table 1:** Inclusion and exclusion criteria

Inclusion criteria	Exclusion criteria
• Age between 50 and 70 years • Women surgically treated for left or right breast cancer and for whom adjuvant treatment is radiotherapy with irradiation of the breast or chest wall irradiation and possibly ganglion chains, • Adjuvant radiotherapy with 3D CRT performed in Clinique Pasteur Toulouse • WHO performance status ECOG (Eastern Cooperative Oncology Group - index usually used to describe the patient's condition) = 0 or 1 • Being volunteer to participate in the study and having signed the consent form	• Indication of adjuvant chemotherapy • Clinically or radiologically detectable metastasis • Personal history of coronary artery disease or myocardial disease • Personal history of breast cancer or other cancer requiring radiotherapy to the thorax • Patient with controlled infection or severe disease and/or non-hazardous to their participation in the study • Contraindications to injection of iodinated contrast (for CCTA): pregnancy, renal failure, allergy • Pregnancy, lactation • Abnormal echocardiography before radiotherapy: - LVEF <50 % - Longitudinal strain > − 16 % - Longitudinal strain rate <1 %/s - Abnormal wall motion • CCTA before radiotherapy showing that therapeutic management is required (by decision of the radiologist and cardiologist)

*3D CRT* 3D conformal radiation therapy, *WHO* World Health Organisation, *CCTA* coronary computed tomography angiography, *LVEF* left ventricular ejection fraction

### Study procedures

**Fig. 1:**
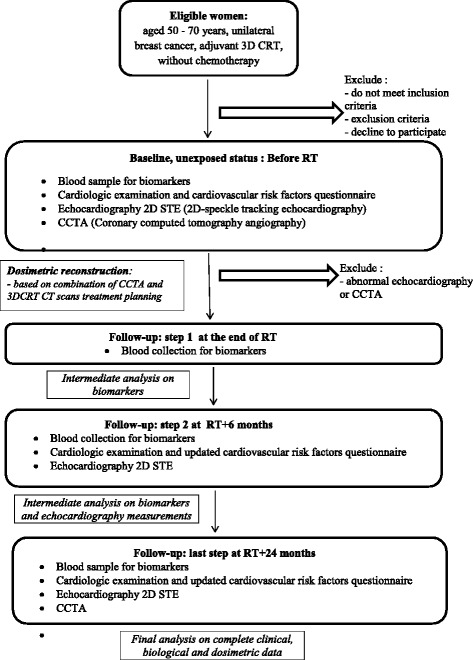
BACCARAT study flowchart

The study flow chart is presented in Fig. 1. It is planned to include the 120 women in 2 years (concordant with the number of patients yearly treated in Clinique Pasteur) and the end of the study is anticipated for the end 2019.

Each patient is included at baseline before RT and followed for 2 years post radiotherapy.

**Fig. 2:**
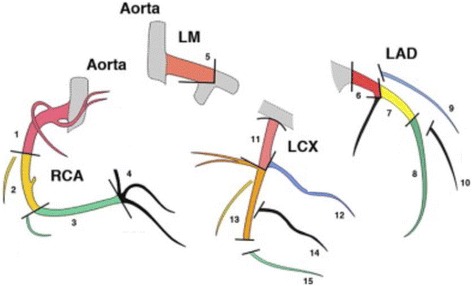
15 segments of coronary arteries. LM: Left main coronary artery; LAD: left anterior descending artery; LCX: left cicumflex artery; RCA: right coronary artery. For each segment, the following index are measures: stenosis in % narrowed; presence of calcification; presence of soft plaque (adapted from [41])

- At baseline, before RT, in addition to classical care, patients have cardiology consultation including description of potential cardiovascular risk factors; 2D-speckle-tracking echocardiography (see Table 2) and Coronary computed tomography angiography (see Fig. 2) are performed; specific blood sample for biomarkers analysis is collected (see the list of biomarkers in Table 3).
- At the end of RT another specific blood sample for biomarkers analysis is collected.
- Six month after RT another specific blood sample for biomarkers analysis is collected and 2D-speckle-tracking echocardiography is performed.
- Two years after RT corresponding to the end of follow-up, a last blood sample for biomarkers analysis is collected; 2D-speckle-tracking echocardiography and Coronary computed tomography angiography are performed.

**Table 2:** Echocardiographic measurements in BACCARAT protocol

- Left Ventricular Ejection Fraction measured using Simpson’s biplane method
- Left ventricular end-diastolic volume measured using Simpson’s biplane method
- Left ventricular end-systolic volume measured using Simpson’s biplane method
- Left ventricular end-diastolic diameter measured using M-mode
- Left ventricular mass measured according to ASE/EAE guidelines
**- Global and segmental longitudinal strain**
**- Global and segmental longitudinal strain rate**
- Global and segmental radial strain rate
- Global and segmental radial strain rate
- E/A wave ratio
- E/Ea wave ratio (lateral annulus)
- TAPSE (tricuspid annular plane systolic excursion)
- Tricuspid annular S wave
- Systolic pulmonary arterial pressure (based on a measure assuming a right atrial pressure of 5 mmHg)
- Left ventricular outflow tract diameter
- Left ventricular outflow tract velocity time integral
- Heart rate
- Cardiac output measured by multiplying heart rate by stroke volume

Bold data are major index taken into account for subclinical cardiac event

**Table 3:** List of circulating biomarkers measured in BACCARAT protocol

Classical biomarkers of cardiac injury
C-reactive protein, Troponin I, B-type natriuretic peptide (NT-Pro BNP), beta2-Microglobulin, Galectin 3
Inflammatory cytokines:
Interleukin 6, Interleukin 8, Interleukin 18, TNF-α
Endothelial activation and dysfunction
sVCAM,-1, s-ICAM-1, E-selectin, P-selectin, vWF, PAI-1, Fibrinogen, Thrombomoduline, TGF-β1
Microparticles
CD14(monocytes), CD31(endothelial), CD41(platelets), CD3(lymphocyte), CD235a (erythrocyte)
microRNAs
miR-1, miR-133, miR-208, miR-499, miR-126, miR-130, miR-145, miR-181, miR-150, miR-155, miR-223, miR-17, miR-18, miR-22, miR-34, miR-92, miR-140, miR-182, miR-199, miR-423, miR-590.

### Study endpoints

The primary endpoint is a subclinical cardiac outcome defined as follows: between baseline (before RT) and 2 years post RT, a decrease in the global and segmental longitudinal strain or strain rate measured by 2D-STE of at least 5 % and/or an increase in the number of coronary segments containing any plaque measured by CCTA of at least 15 %. These average percentages were chosen based on those observed in previous studies and can be considered clinically pertinent [17, 34–37].

The secondary endpoints are:

- Decrease in the strain or strain rate between measurements made before RT and 6 months after RT.
- Modified series of biomarkers between the measurements before RT and: just after RT, 6 months after RT, 24 months after RT.
- Correlation between the absorbed radiation dose to the whole heart and different structures of the heart and measurements of strain and strain rate and number of coronary segments containing any plaque measured 2 years after RT.

### Sample size calculation

The sample size was based on a statistical power of 80 %, an alpha-risk of 5 %, the definition of the primary endpoint (a decrease in the global and segmental longitudinal strain or strain rate of at least 5 % and/or an increase in the number of coronary segments containing any plaque of at least 15 %). Baseline measurements were collected from previous publications:

- Mean global longitudinal strain before RT [36]: −16.5 % ± 2.1 %.
- Mean global longitudinal strain before RT [36]: −1.00/s ± 0.13/s.
- Number of segments with coronary plaques [37]: 2.1 ± 1.0 segments.

Taking into account tailed test for comparisons, but also exclusion criteria (±10 % of women whose echocardiography or CCTA at baseline may be abnormal and require therapeutic management and should be excluded; ± 5 % of women may resort to cardiotoxic chemotherapy during the 2 year follow-up and should also be excluded; less than 1 % of women likely to die within 2 years after RT - these women will be analyzed until the date of death), the inclusion of 120 women is necessary.

### Planned analysis

A strength of the study is that each patient will her own control (unexposed status before RT) and evaluations will be based on individual changes of measurements over time. Paired tests for comparisons will be used. All tests will be bilateral with alpha = 5 %. Patients’ baseline characteristics before RT (pre-RT) will be used as "reference" values.

- (o) Description of the population at baseline (before RT) will be performed including: the medical data collected in the questionnaire, in particular the risk factors for cardiovascular disease; measurements of all biomarkers; 2D-STE measurements including strain and strain rate; CCTA measurements including mean number of coronary segments with plaques. Descriptive statistics (means, standard deviations, percentages and confidence intervals of 95 %) will be used the whole cohort.
- (i) For the analysis of primary endpoint, the occurrence and progression of early subclinical cardiac lesions will be analyzed based on prevalence study and Chi-square tests.
- (ii) Wilcoxon tests for paired-samples will be used to study the variations of targeted circulating biomarkers during follow-up. Crude temporal trends will also be considered.
- (iii) A detailed dose reconstruction will be performed for each woman. CT images used for treatment planning allow delineation of the heart and surrounding organs. However, on these images the coronary arteries are not visible and cannot be delineated. Registration of the planning and coronary angiography CT images will allow delineation of the coronary arteries on the planning CT images. Using the 3D dose matrix associated with the RTDose file generated during treatment planning [38] and the added coronary contours, dose volume histograms and 3D dose maps [28] of the whole heart and coronaries will be generated. This process will be performed with the Isogray treatment planning software (http://www.dosisoft.com/en/rt-planning/tps-isogray/dose-calculation-models.html). A description of the doses thus obtained will be shown for the whole cohort of women.
- (iv) Spearman correlations will provide results on the association between measurements of strain and strain rate and indices of coronary plaques and the measurement of biomarkers and the radiation dose absorbed by the different structures of the heart. Dose-response relationship between subclinical and dosimetric data will be studied in order to provide results on the effect of dose on subclinical outcome. These relationships will be established from coarse to fine levels, considering first the heart as a whole and going through different substructures. Logistic regression models as well as generalized linear model and repeated data models could be used for the study of the variation between 0 and 2 years the intermediate measurements.
- (v) For individualized risk for each patient, multivariate model of normal tissue complication probability (NTCP), which takes into account relationships among all parameters will be proposed based on subclinical cardiac lesions occurrence, including clinical, biological and dosimetric data using logistic regression and forward variable selection.

## Discussion

Breast RT was associated with long-term cardiac toxicity more than 10 years after treatment with relative risks of clinically significant cardiac events, including ischemic heart disease, within the range of 1.2 to 3.5. In France, ischemic heart disease is the first specific cause of death among women and breast cancer. It is the most frequent type of cancer among women with nearly 50,000 new cases diagnosed each year. BACCARAT is thus positioned at a crossing point of two leading causes for women morbidity.

Today, some residual risk of secondary effects due to the presence of normal tissues in the irradiation field remains. This can unfortunately affect the quality of life of breast cancer survivors, who are increasingly frequent. As a consequence, there is a need for further research to improve early detection of late cardiac effects in mostly asymptomatic patients, and also to improve prediction and prevention [39].

The BACCARAT project is a multidisciplinary novel approach to early detect radiation-induced cardiotoxicity based on an early stage clinical study. The long term significance of the observed changes being an important issue, at the end of the 2-year follow up of the study, each patient will be proposed to participate in a large multicentric study on long term follow-up of cardiac events with clinical follow-up going forward for 10 years at least.

Some hypothesis will be investigated in BACCARAT: the choice of subclinical cardiac lesions index, the supervised analysis of targeted biomarkers, the methodology and clinical application for a precise heart dosimetry and doses constraints that could be enhanced during RT, the choice to model and possibly predict the cardiotoxicity risk by combining biological, clinical and dosimetric parameters.

The dosimetric work performed in BACCARAT with a degree of precision never reached before is a strength of the study. Effects of specific doses to the whole heart and to specific cardiac substructures have only been assessed in a few studies [4] [30] that revealed the importance of better knowledge for the effects of radiotherapy to critical structures of the heart, including the effect of both radiation dose and volume of the heart exposed. In particular, based on dose reconstructions performed with patient-specific simulation CT scan and CCTA, Moignier et al. [30] showed that use of mean heart dose as surrogate to the coronary doses is not reliable. This patient-specific approach was retained for BACCARAT dosimetry. Consideration of irradiated structures within the heart may prove fruitful for the future and is concordant with recommendations for future studies based on radiation dosimetry that are able to take into account the distribution of dose within the heart (rather than just the mean heart dose) may provide a better prediction of the heart diseases following breast radiotherapy [40, 41]. Moreover, the approach of simultaneous assessment of multiple biomarkers, including microparticules and miRNAs was never performed before and should help to understand some biological mechanisms involved in the radiation-induced cardiac changes.

Provided with the patient’s own biological, clinical and dosimetric parameters, the NTCP models should allow an individualized risk for each patient. These new tools and results developed in the BACCARAT project will allow for the optimization of radiotherapy protocols leading to personalized treatments with increased therapeutic efficacy. It should also improve prediction and prevention of potential lesions to cardiac normal tissues surrounding tumors and ultimately enhance patients’ care and quality of life.

BACCARAT project could finally enhance cardio-oncology that has developed recently to ensure the implementation of primary prevention strategies and screening protocols for early recognition of cardiotoxicity in RT treated patients, as early administration of advanced treatment for signs of cardiac lesions may be crucial. By anticipating the cardiac risk after breast RT, one could reduce the cost for patients care.

### Ethics approval and consent to participate

This study has received ethical approval from the French South West Committee for Protection of Persons (ID: CPP2015/66/2015-A00990-69) and from National Agency for Medical and Health products Safety (Reference: 150873B-12). Participants enrolled in the study provide their written informed consent.

### Consent for publication

Not applicable.

### Study registration

The study is registered on clinicaltrials.gov with the following ID: NCT02605512.

### Status of the study

The study is currently recruiting patients.

## Abbreviations

2D STE: 2D speckle-tracking echocardiography
3D CRT: 3 dimensional conformal radiation therapy
CCTA: Coronary computed tomography angiography
LVEF: left ventricular ejection fraction
NTCP: normal tissue complication probability
RT: radiotherapy
